# Lung cancer surgery with partial anomalous pulmonary venous connection presenting an inverted Scimitar sign

**DOI:** 10.1186/s44215-026-00245-6

**Published:** 2026-03-07

**Authors:** Koki Maruyama, Mitsuhiro Isaka, Daisuke Yamaguchi, Keigo Matsusima, Tatsuya Masuda, Momoko Asami, Kazuki Hayasaka, Sinya Katsumata, Hayato Konno, Hideaki Kojima, Naoya Yokomakura, Yasuhisa Ohde

**Affiliations:** Division of Thoracic Surgery, Shizuoka Cancer Center, 1007 Shimonagakubo, Nagaizumi-Cho Sunto-Gun, Shizuoka, 411-8777 Japan

**Keywords:** Partial anomalous pulmonary venous connection, Partial anomalous pulmonary venous return, Lung cancer

## Abstract

**Background:**

Partial anomalous pulmonary venous connection (PAPVC) is a congenital anomaly in which a part of the pulmonary veins drain into the right heart system. Many cases of PAPVC remain asymptomatic and often do not present clinical problems. However, when performing a lung resection, it becomes important in terms of surgical techniques and complications.

**Case presentation:**

A 70-year-old woman was referred to our hospital for evaluation of an abnomal shadow on chest radiography performed during medical check-up. A contrast-enhanced chest computed tomography (CT) revealed a part-solid nodule in the right upper lobe (RUL). Additionally, PAPVC was observed, in which almost all pulmonary veins, except for a small part of the basal segmental pulmonary vein (PV), drained into the azygos vein. The patient was diagnosed with suspected primary lung cancer (cT1miN0M0, stageⅠA1) with PAPVC. We performed righit upper wedge resection while preserving the PAPVC.

**Conclusion:**

We reported rare PAPVC presenting an inverted Scimitar sign. To the best of our knowledge, this is the first case report of its kind. The tumor was located very close to the main trunk of the anomalous PV. Resection of the main trunk would have resulted in the loss of all pulmonary veins except for the basal segmental PV. Furthermore, the surgical margin would have been unchanged when comparing right upper lobectomy with wedge resection. Therefore, wedge resection of the RUL was selected. The resection margins were negative.

**Supplementary Information:**

The online version contains supplementary material available at 10.1186/s44215-026-00245-6.

## Background

PAPVC is a congenital anomaly in which a part of the pulmonary veins drain into the right heart system. Many cases of PAPVC remain asymptomatic and often do not present clinical problems. Surgical repair of PAPVC is recommended in patients with symptoms and/or an elevated pulmonaly-to-systolic blood flow ratio (Qp/Qs). When pulmonary resection is scheduled in patients with lung tumor and PAPVC, not only Qp/Qs, but also the location of the PAPVC and lung tumor is important. We performed lung surgery with rare form of PAPVC located in the ipsilateral to the tumor.

## Case presentation

**Fig. 1:**
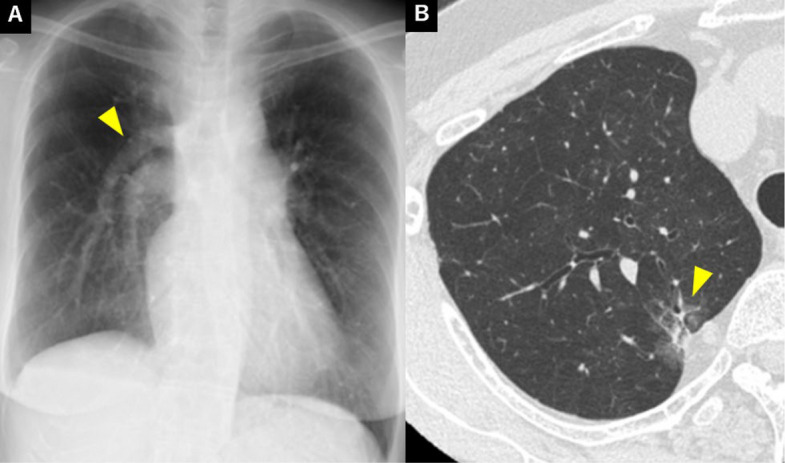
**A** Chest X-ray. An abnomal pulmonary venous shadow in the rigt lung field. **B** Chest CT. A 25 mm part solid nodule was detected in the RUL

**Fig. 2:**
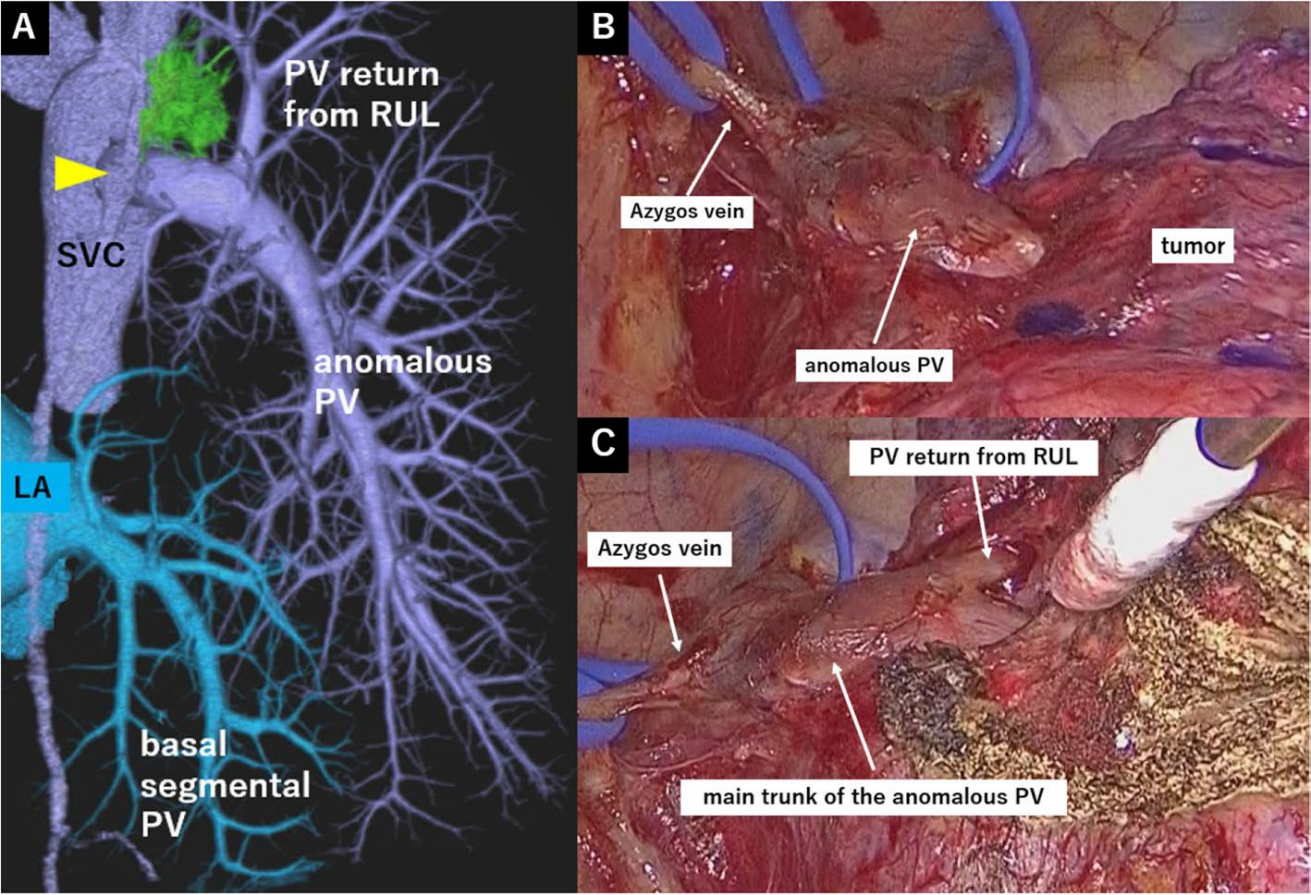
**A** 3D-CT Angiography. Except for only a few basal segmental PV, most pulmonary veins returning to the azygos vein (arrow). **B** Perioperative image. Anomolous PV drained into the azygos vein. **C** The tumor is located closer to the main trunk of the anomalous PV than to the PV return from RUL

A 70-year-old woman, who never smoked, was referred to our hospital for evaluation of an abnomal shadow on chest radiography performed during medical check-up. Routine laboratory tests were within the nomal range. A chest X-ray showed vascular shadow running parallel to the pulmonary artery shadow (Fig. 1A). A contrast-enhanced chest CT revealed a part-solid nodule in the RUL, measuring 2.5 cm in total diameter with a solid component of 0.5 cm (Fig. 1B). Additionally, PAPVC was observed, in which almost all pulmonary veins, except for a small part of the basal segmental PV, drained into the azygos vein (Fig. 2A). 18-fluorine-fluorodeoxyglcose positron emission tomography/computed tomography (18F-FDG PET/CT) revealed FDG accumulation only in the nodule (maximum standardized uptake value 2.49), we could not detect any lymph node metastasis or distant metastasis. The patient was diagnosed with suspected stage ⅠA1 lung cancer. Pulmonary function tests showed no remarkable findings. Echocardiography showed no evidence of atrial septal defect or other abnormalities. The tumor was located very close to the main trunk of the anomalous PV, and resection of the main trunk would have resulted in the loss of all pulmonary veins except for a small part of the basal segmental PV.　Furthermore, the surgical margin would have been unchanged when comparing right upper lobectomy with wedge resection. Moreover, based on the imaging findings, the tumor was deemed low-grade malignancy. Therefore, wedge resection of the RUL was selected. Under general anesthesia and double-lumen intubation, we performed partial resection of the RUL. The lung showed absence of interlobar fissures, and the main pulmonary venous trunk drained into the azygos vein (Fig. 2B). First, we dissected the lung parenchyma from the main trunk of the PAPVC. During this procedure, a small branch of the PV from the RUL was divided. To preserve the main branch from RUL to the trunk of PAPVC, which corresponds to the superior pulmonaly vein in nomal anatomy, it was necessary to extend the distance between the vessel and the tumor. After confirming the branch from RUL, lung parenchymal resection was performed between the right upper and lower lobes using electrocautery. To mobilize the tumor dorsally, the resection of the lung parenchyma was extended ventrally while identifying the branch from the RUL. This approach enabled a stapled wedge resection while preserving both the branch and the main trunk of the PAPVC [see Additional file 1]. The chest drain was removed on postoperative day 1, and the patient postoperative course was uneventful. Postoperative pathological examination confirmed lepidic adenocarcinoma, classified as pT1aN0M0, Stage IA (maximal diameter 8 mm). The resection margins were negative (margin length 7 mm). The patient was followed up without any complications.

## Discussion

Anomalous pulmonary venous connection is a congenital anomaly in which pulmonary veins drain into the right atrium or systemic veins. It is classified into total anomalous pulmonary venous connection (TAPVC), in which all pulmonary veins drain abnormally into the right atrium, and PAPVC, in which only some pulmonary veins are abnormally connected while the rest drain normally into the left atrium. Drainage sites and frequency of PAPVC is superior vena cava (35.8%), left brachiocephalic vein (20.6%), right atrium (19.6%), left superior vena cava (7.6%), inferior vena cava (4.3%), azygos vein (3.3%), others (8.8%) [1]. A PubMed search using the keywords 'anomalous pulmonary venous connection or return' and 'lung cancer' identified 8 reported cases of PAPVC draining into the azygos vein [2–9]. The abnormal pulmonary veins in each case were as follows: in 7 cases, a part of the right superior PV was involved; and in 1 case, the left inferior PV was involved. In our case, all pulmonary veins except for a small part of the basal segmental PV drained into the azygos vein, showing a much more extensive anomaly compared to previously reported cases. In lung resection with coexisting PAPVC, the anatomical relationship between the anomalous pulmonary veins and the tumor is critically important. When PAPVC is present in a non-resected lobe, there may be an increase in shunt volume postoperatively, which can lead to right heart failure. Therefore, it is necessary to assess the Qp/Qs using a preoperative right heart catheterization to evaluate the need for revascularization. Additionally, there are reports of cases in which vascular resection was performed after confirming the absence of hemodynamic changes during intraoperative PV occlusion testing [5]. Black et al. [10] reported a case of right heart failure that occurred after a right pneumonectomy. PAPVC of the left superior PV was later diagnosed by right heart catheterization, necessitating vascular reconstruction. However, the patient died on postoperative day 18. In cases of extensive lung resection, particular attention should be paid to a contralateral PAPVC relative to the planned side of surgery. In our case, due to the anatomical relationship between PAPVC and the tumor, a relative increase in shunt volume did not occur. Scimitar syndrome is a rare form of PAPVC, characterized by most of the right pulmonary veins draining into the inferior vena cava. The name 'Scimitar syndrome' derives from the large abnormal vein similar to a curved Turkish sword (a scimitar) on radiography. Scimitar sign is a curved, tubular opacity adjoining to right of heart and reaching towards the diaphragm. In our case, the chest X-ray findings resembled an inverted Scimitar sign. Due to this characteristic appearance, the anomaly had been noted during previous routine health checkups. Yuki et al. [11] reported a case of Scimitar syndrome with a left metastatic lung tumor. In that case, vascular reconstruction was performed before lung surgery, involving a sternotomy and right atriotomy, followed by fenestration of the fossa ovalis to redirect right pulmonary venous blood to the left atrium. In conclusion, we report a case of lung cancer resection with rare PAPVC presenting an inverted Scimitar sign. Partial resection was performed safely without postoperative cardiac and respiratory complications. It is important to perform preoperative imaging analysis with careful attention to PAPVC, including evaluation of the contralateral lung.

## Supplementary Information

Additional file 1.

## Data Availability

Not applicable.

## Abbreviations

PAPVC: Partial anomalous pulmonary venous connection
PV: Pulmonary vein
CT: Computed tomography
RUL: Right upper lobe
Qp/Qs: Pulmonaly-to-systolic blood flow ratio
